# Involvement of the STING signaling in COVID-19

**DOI:** 10.3389/fimmu.2022.1006395

**Published:** 2022-12-08

**Authors:** Ruoxuan Xiao, Ao Zhang

**Affiliations:** ^1^ Research Center for Small Molecule Immunological Drugs, School of Pharmaceutical Sciences, Shanghai Jiao Tong University, Shanghai, China; ^2^ Pharm-X Center, Laboratory of Medicinal Chemical Biology & Frontiers on Drug Discovery (RLMCBFDD), School of Pharmaceutical Sciences, Shanghai Jiao Tong University, Shanghai, China

**Keywords:** COVID-19, SARS-CoV-2, STING, vaccine, IFNs

## Abstract

The coronavirus disease 2019 (COVID-19) pandemic caused by the infection of severe acute respiratory syndrome coronavirus 2 (SARS-CoV-2) has cast a notorious damage to the public health and global economy. The Stimulator of Interferon Genes (STING) is a crucial element of the host antiviral pathway and plays a pivotal but complex role in the infection and development of COVID-19. Herein, we discussed the antagonistic mechanism of viral proteins to the STING pathway as well as its activation induced by host cells. Specifically, we highlighted that the persistent activation of STING by SARS-CoV-2 led to abnormal inflammation, and STING inhibitors could reduce the excessive inflammation. In addition, we also emphasized that STING agonists possessed antiviral potency against diverse coronavirus and showed adjuvant efficacy in SARS-CoV-2 vaccines by inducing IFN responses.

## Introduction

Coronavirus disease 2019 (COVID-19) has rapidly spread across the world since the end of 2019, resulting in over 500 million confirmed infected cases and over 6 million deaths so far (https://www.who.int/). The corresponding virus of COVID-19 was identified as severe acute respiratory syndrome coronavirus 2 (SARS-CoV-2) virus, which contains a positive single-stranded RNA genome (1, 2). COVID-19 has caused disastrous damage to the public health and the economic development of the world, and a few treatment options and vaccines have been developed to reduce it (3, 4). Remdesivir, a broad-spectrum antiviral drug, is the first drug approved by FDA for COVID-19 treatment (5). Another small molecule antiviral agent, Paxlovid (nirmatrelvir/ritonavir), was approved for the treatment of adult patients with mild-to-moderate COVID-19 (6). Meanwhile, oral or intravenous administration of dexamethasone was reported to reduce the 28-day mortality in patients hospitalized with COVID-19 (7). Additionally, various therapeutic monoclonal antibodies have been applied to treat COVID-19, such as Regkirona (regdanvimab) and REGEN-COV (casirivimab and imdevimab) (8–10). Combination of interleukin-6 receptor blocker (tocilizumab or sarilumab) and JAK inhibitor baricitinib is strong recommended for patients with severe or critical COVID-19 (11). Moreover, a number of vaccines has been developed to prevent the infection of SARS-CoV-2, such as BNT162b2 and mRNA-1273, demonstrating appreciable efficacy in phase III clinical trials (12, 13). Nevertheless, available drugs and vaccines are insufficient to combat the continuous emergence of viral variants, and the excessive inflammation induced by existent treatments should be concerned. Therefore, it is urgent to develop novel prophylactic and therapeutical measures to prevent SARS-CoV-2 from continuous infection, mutation, and transmission.

**Figure 1:**
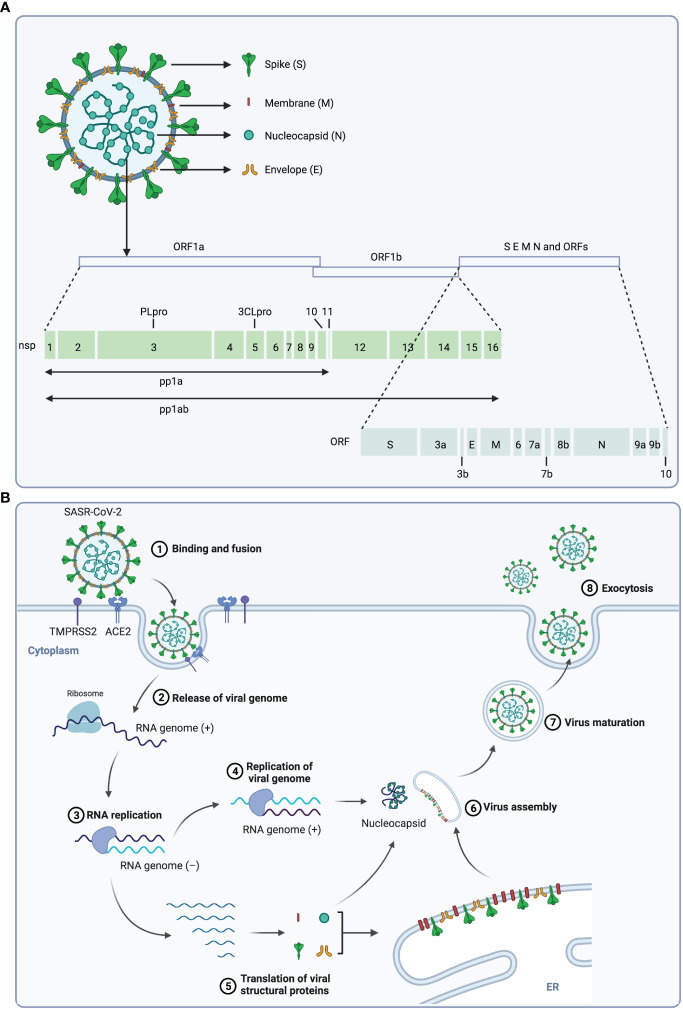
The structure and life cycle of SARS-CoV-2 virus. **(A)** The structure and genome of SARS-CoV-2 virus. The SARS-CoV-2 virus is composed of four structural proteins (spike protein S, membrane protein M, envelope protein E, and nucleocapsid protein N) and a single-stranded RNA genome. The virus genome contains 14 ORFs encoding 16 nonstructural proteins, 4 structural proteins and 9 accessory proteins respectively. **(B)** Scheme of the SARS-CoV-2 replication cycle. At the initial step of infection of this virus, the S1 subunit of the S protein interacts with the receptor angiotensin-converting enzyme 2 (ACE2) of host cells, and the S2 subunit is cleaved by TMPRSS2, a serine protease on the host cell surface, to promote uptake and fusion. Subsequently, the viral RNA is released into the cytoplasm of the host cell, and the ORF1a and ORF1b at the 5’-end are translated to polyproteins (pp1a and pp1ab), which is then cleaved by viral proteases 3CLpro and PLpro to 16 nonstructural proteins (nsps). These nsps form the replication and transcription complex to synthesize progeny viral genomic RNA. In parallel, ORFs at 3’-end are translated to structural proteins, and the S, M and E proteins are translocated to the ER-to-Golgi compartment, where they are assembled with N-encapsulated genomic RNA and then secreted out of cell through exocytosis.

SARS-CoV-2 belongs to the β-coronavirus genus, which includes SARS-CoV, Middle East respiratory syndrome (MERS)-CoV and bat coronavirus HKU4 and so on (14–17). The virus genome consists of 14 open reading frames (ORFs) that encode 16 nonstructural proteins (nsp), structural proteins (spike protein S, membrane protein M, envelope protein E, and nucleocapsid protein N) and 9 accessory proteins (ORF3a, ORF3b, ORF6, ORF7a, ORF7b, ORF8, ORF9b, ORF9c and ORF10) (**Figure 1A**) (1, 18). The life cycle of SARS-CoV-2 is displayed in **Figure 1B**, and the interaction between the S protein of SARS-CoV-2 and the ACE2 on host cells is essential for the infection, thus ACE2 and S protein are important targets for treatment of COVID-19 (18, 19).

The innate immune system is the first line of defense against evading pathogens (20). It recognizes pathogen/damage associated molecular patterns (PAMPs/DAMPs) by pattern recognition receptors (PPRs), including Toll-like receptors (TLRs), Nod-like receptors (NLRs), RIG-I-like receptors (RLRs) and the DNA sensor cyclic guanosine monophosphate (GMP)-adenosine monophosphate (AMP) synthase (cGAS)-stimulator of interferon genes (STING) signaling pathway. Among them, the cGAS-STING pathway plays an important role in innate immune response to pathogen infection. Mechanistically, the double stranded DNA (dsDNA) of pathogens is accumulated in cytoplasm and activates cGAS to generate 2′3′-cyclic GMP-AMP (2′3′-cGAMP), which binds to and activates STING (21–24). The bound STING is translocated from endoplasmic reticulum (ER) to Golgi, where it recruits the kinase TANK-binding kinase 1 (TBK1) and stimulate IκB kinase (IKK), causing phosphorylation of interferon regulatory factor 3 (IRF3) and nuclear factor-κB (NF-κB) (25, 26). Subsequently, the transcription of type I interferons (IFNs) and other inflammatory genes was triggered, which mediate immune response to eliminate pathogens (27–29). In addition to dsDNA from pathogens, endogenous DNA including chromosomal DNA and mitochondrial DNA can also trigger the cGAS-STING signaling pathway. Normally, chromatin is strictly compartmentalized in the nucleus to prevent cGAS-STING activation, while chromosome mis-segregation during cell mitosis leads to the generation of micronuclei, induing the aberrant recognition by cGAS (30, 31). Similarly, abnormal packaging of mitochondrial DNA (mtDNA) facilitates the escape of mtDNA into cytosol, which induces the activation of cGAS-STING (32).

As an RNA virus, SARS-CoV-2 is primarily recognized by RLRs in the host cells (33). Interestingly, increasing evidences demonstrated that the cGAS-STING pathway, a key DNA sensor, restricted the infection of RNA virus, and the proteins of RNA virus could antagonize the cGAS-STING signalling (34). For example, *Sting* ^-/-^ mice were more sensitive to vesicular stomatitis virus (VSV), a negative-stranded virus, and the production of type I IFNs was decreased in *Sting* ^-/-^ mice (35). Besides, the papain-like protease (PLpro) of SARS-CoV was reported to disrupt the STING-tumor necrosis factor receptor-associated factor 3 (TRAF3)-TBK1 complex by directly binding to it, and the dimerization and ubiquitination of STING were blocked by the PLpro of SARS-CoV and human coronavirus (HCoV) (36, 37). Hence, as an RNA virus, how SARS-CoV-2 interacts with STING pathway is worthy of further exploration.

## SARS-CoV-2 regulates STING signaling

SARS-CoV-2 infection has a double-edged effect on the STING signaling, dependent on the stage of disease procession and the infected tissues. Initially, Rui and colleagues hypothesized that SARS-CoV-2 might antagonize the innate immune pathway due to the antiviral function of STING (38). They investigated the effect of SARS-CoV-2 proteins on STING and RLR-mediated immune response, and found that both ORF3a and 3CL of the virus could inhibit STING and the downstream NF-κB signaling, but not IRF3 signaling, and this process was independent of cGAS. Further, it was found that ORF3a directly interacted with both the N-terminal and the C-terminal fragment of STING and suppressed the nuclear accumulation of p65, which then inhibited STING-mediated NF-κB signaling. While viral 3CL, through its enzymatic activity, inhibited the NF-κB pathway by suppressing the K63-ubiquitination of STING. In addition, the polymorphisms of STING from different species, including human, mouse and chicken could be inhibited by 3CL and ORF3a. However, bat STING, the natural host of SARS-CoV-2, was found defective to produce type-I interferon (IFN) and thus showed compromised anti-viral potency (39, 40). These results suggest that STING might be involved in the transmission of the virus. This is the first study supporting that SARS-CoV-2 can suppress STING signaling to escape from innate immune response.

Similarly, Han and co-workers reported that the ORF9b of SARS-CoV-2 suppressed the induction of type I and III interferons through multiple innate immune signaling, including RLR, TLR and STING (41). The antiviral activity of the host cell against SARS-CoV-2 depends on the production of type I and III IFNs, which is impaired in the serum of COVID-19 patients. However, the ORF9b of SARS-CoV-1 has been reported to inhibit IFNs response (2, 42–44). Based on these findings, they explored the effect of ORF9b on IFNs response and found that ORF9b of SARS-CoV-2 antagonized type I and III IFN responses induced by SeV and suppressed the activation of RLR, TLR and the cGAS-STING pathway. Mechanistic studies indicated that ORF9b directly interacted with RIG-I, MDA-5, MAVS, TBK1, TRIF and STING, and suppressed the phosphorylation of TBK1 and IRF3 along with IRF3 nuclear translocation. Furthermore, overexpression of viral ORF9b facilitated VSV infection, suggesting that ORF9b is closely implicated with the pathogenesis of COVID-19.

Recently, the ORF10 of SARS-CoV-2 has also been found to antagonize STING-dependent interferon response. Han et al. screened 29 SARS-CoV-2 viral proteins, and found ORF10 could suppress the activation of the cGAS-STING pathway by interacting with STING directly. As a result, the STING-TBK1 interaction was impeded, and the translocation of STING was blocked, leading the immune evasion of SARS-CoV-2 (45).

Taken together, various components of SARS-CoV-2 could inhibit the STING pathway and subsequent interferon response, leading to virus escape from innate immunity (**Table 1**).

**Table 1:** The effect of SARS-CoV-2 viral components on the STING signaling pathway.

Viral components	Interaction with STING	Mechanism of action	Ref
**ORF3a**	Directly bind with STING	Inhibit the nuclear accumulation of p65 to inhibit the NF-κB pathway	(38)
**3CL**	Inhibit the K63-ubiquitination of STING	Inhibit the NF-κB pathway and the recruitment of TBK1 and IKKβ	(38)
**ORF9b**	Directly bind with STING	Inhibit the phosphorylation of TBK1 and IRF3 as well as the nuclear translocation of IRF3	(41)
**ORF10**	Directly bind with STING	Impair the STING-TBK1 interaction and STING translocation	(45)

Intriguingly, contrary to the aforementioned findings that SARS-CoV-2 antagonizes the STING signaling, many other studies suggest that infection with SARS-CoV-2 could trigger STING signaling. Transcriptome data showed that at the time of diagnosis, the content of STING protein was increased in the blood of patients with mild or moderate symptoms, whereas there is no significant change in the severe patients (46). Furthermore, STING expression was found to be elevated only in moderate patients at a few days after diagnosis (46). These data indicate that activation of STING might be associated with the severity and stage of COVID-19.

More detailed mechanistic studies indicate that cell fusion and formation of syncytia and micronuclei play crucial roles in SARS-CoV-2-induced cGAS-STING signaling (47, 48). Previous study has demonstrated that cell fusion mediated by the interaction of S protein with host ACE2 results in the formation of syncytia, presenting as a single cell containing several nuclei (49). Further, Ren and co-workers explored molecular events after syncytium formation in the well-established syncytia model. The results showed that both S protein and SARS-CoV-2 induced syncytium formation in HeLa-ACE2 cells and then led to production of micronuclei. Eventually, DNA damage and genome instability of the micronuclei promoted the activation of the cGAS-STING pathway as well as the downstream IFN response (50).

Furthermore, more studies were conducted to elucidate the mechanism of innate immune activation caused by SARS-CoV-2 infection. In addition to the activation of cGAS-STING induced by DNA from micronuclei, Zhou and co-workers further demonstrated that syncytia formation caused cytoplasmic chromatin by disrupting the actin cytoskeleton and nuclear lamin A/C, which are important factors for maintaining nuclear morphology. Meanwhile, they found that STING agonists (diABZI and SR-717) exhibited antiviral activity against SARS-CoV-2 (51). Meanwhile, cleavage of S protein by host proteases was found essential for cell fusion and IFN response (52).

The generation of syncytia provides a possible mechanism for delayed IFN response in COVID-19 patients, indicating that the production of type I IFN is inhibited at early stage of SARS-CoV-2 infection, but then substantially activated at the late stage (44, 53).

## The aberrant inflammatory response caused by STING activation

Although STING activation presents antiviral potential, the sustained STING signaling results in excessive amounts of type I IFNs. Indeed, patients with severe COVID-19 exhibited robust type I interferon response, which was associated with acute respiratory distress syndrome, lung injury and poor clinical outcome (1, 54–57). Therefore, over-activation of STING pathway may lead to hyperinflammation and related syndromes in COVID-19 patients.

By profiling the transcriptome and secreted cytokines of SARS-CoV-2-infected lung epithelial cells, Neufeldt and co-workers found that the NF-κB and pro-inflammatory pathway was up-regulated in infected lung epithelial cells, while the antiviral IFN response was not enhanced (58). These observations were consistent with the clinical features of severe patients (2, 59). Subsequent studies further proved that the hyper-inflammatory response is attributed to the activation of NF-κB but not IRF3, and is mediated by cGAS-STING pathway rather the RLR and TLR pathways. This imbalanced immune response recruited macrophage and neutrophils to cause cell death and lung pathology (58, 60). Finally, VS-X4 and H151, the established STING inhibitors (61), suppressed the upregulation of inflammatory cytokines and alleviated the abnormal immune response, which may protect COVID-19 patients from further suffering of this disease (62, 63).

Different from other studies on lung epithelial cells or tumor cell models, Domizio and colleagues focus on skin manifestations in SARS-CoV-2 infection (64). They found that cGAS-STING signaling and subsequent type I IFN production were initiated in endothelial cells and perivascular macrophages around injured vessels. As a result, the production of type I IFN in endothelial cells promoted cell death and tissue damage. Accordingly, administration of the STING inhibitor H151 reduced type I IFN response and related lung pathology in mice infected with SARS-CoV-2 (19).

## STING agonists inhibit SARS-CoV-2

Since viral proteins suppress the STING pathway in the early stage of infection, while the micronucleus and DNA damage caused by cell fusion in the host activate the STING signaling to suppress viral infection, treatment with STING agonists in the early stage of COVID-19 provides be a potential antiviral strategy.

Recently, Li and co-workers found that SASR-CoV-2 infection induced delay of IFN response to evade innate immunity, which could be controlled by type I IFN treatment (65). Subsequently, they screened 75 agonists targeting diverse PRR pathway and identified cyclic dinucleotides (CDNs), the endogenous stimulator of STING (66), showing antiviral activity against SARS-CoV-2. One of the potent STING agonists diABZI was then tested in subsequent studies due to its significant potency and higher bioavailability. As expected, diABZI elicited potent and transient innate signaling and prevented SARS-CoV-2 infection in primary human respiratory epithelial cells as well as the lung of mice. A single intranasal delivery of diZBAI protected mice from lethality induced by SARS-CoV-2 and its South African variant B.1.351, thus supporting the therapeutic potential of diABZI against diverse trains of SARS-CoV-2 (65).

Similarly, the diABZI analogue, diABZI-4, was proved as well to prevent SARS-CoV-2 replication in ACE2-A549 cells and in 3D-cultured embryonic stem cell–derived induced alveolar type II (iAT2) cells (67). Intranasal administration of diABZI-4 before or after virus infection reduced weight loss and death in K18-ACE2 mice without pathological damage in lung tissues. Furthermore, diABZI induced transient pro-inflammatory cytokines production and promoted the activation of myeloid cells, T cells and NK cells, without pathological damage and excessive inflammation in lung tissue (67).

Taken together, STING agonists could effectively activate the antiviral response and prevent SARS-CoV-2 infection *in vivo* and *in vitro*. The activation is transient, but can prevent lung tissue damage from abnormal inflammation. Therefore, STING agonists may provide alternative strategy for the treatment of COVID-19 in the early stage (**Table 2**).

**Table 2:** The effect of STING inhibitors and agonists on SARS-CoV-2.

Catalogue	Compound	Model	Effect	Mechanism of action	Ref
Inhibitor	VS-X4	Calu-3 cells and A549-ACE2 cells	Limit SARS-CoV-2 mediated inflammation	Decrease TNF, IL-6 and IP-10 upregulation caused by infection; decrease p65 nuclear accumulation	(58)
	H-151	Calu-3 cells and A549-ACE2 cells	Limit SARS-CoV-2 mediated inflammation	Decrease TNF upregulation caused by infection; decrease p65 nuclear accumulation	(58)
	H-151	Lung-on-chip (LoC) model; K18-hACE2 transgenic mice	Reduce inflammatory cell infiltration; attenuate lung injury, weight loss and mouse death after infection	Reduce ISGs expression and cytopathic effect induced by infection in endothelial cells	(64)
Agonist	diABZI	MucilAir™ reconstituted from human bronchial biopsies primary cells	Inhibit SARS-CoV-2 infection; prevent epithelial damage	/	(68)
	diABZI	A549-ACE2 cells; human lung tissue slices	Inhibit SARS-CoV-2 infection	/	(69)
	diABZI	Primary NHBE cells; K18-hACE2 transgenic mice	Decrease viral replication, weight loss and lung inflammation	Transiently stimulate IFN signaling and mildly induce TNFα; activate JAK-STAT signaling; decrease immune cells in lung	(65)
	diABZI	Calu-3 cells and HeLa-ACE2 cells	Inhibit SARS-CoV-2 replication	Increase the phosphorylation of IRF3	(51)
	diABZI-4	A549-ACE2 cells; Embryonic stem cell–derived induced alveolar	Inhibit SARS-CoV-2 gene expression and replication; prevent SARS-CoV-2 infection; alleviate wight loss	Induce oligomerization of STING and ISGs expression; activation of myeloid cells, γδT cells, and NK cells	(67)

## STING agonists as adjuvant of SARS-CoV-2 vaccine

Vaccines have made great contribution to COVID-19 prevention (70). Of which subunit vaccines are the most used due to their excellent efficacy and safety. The spike protein of SARS-Cov-2 and its receptor binding domain (RBD) have been considered as the two main antigens in COVID-19 vaccine, because their corresponding epitope domains could induce the production of neutralizing antibodies (71, 72). However, the poor immunogenicity of the highly purified S protein and RBD limits the development of effective SARS-CoV-2 vaccine. Therefore, additional adjuvants are necessary to elicit robust and durable immune response. Aluminum salts (Alum) are the most commonly used adjuvant, however, poor antibody immune response and predominant Th2 response restrict their use for various antigens (73, 74). With the development of innate immunity research, PAMPs/DAMPs has attracted attention as potential vaccine adjuvants, and STING agonists has been employed as adjuvants in multiple pre-clinical vaccines (75). Mechanistically, activation of STING could maturate DCs and prime T cells, leading to subsequent humoral immunity to control virus (76, 77). Neutralizing antibodies produced by humoral immunity contributes to the virus clearance potency of vaccines, and STING agonists were reported to increase antibody titers and trigger potent humoral immune response (78).Additionally, STING agonists induced the formation of germinal center (GC), where B cells were primed and differenced into memory B cells to achieve long-lasting profection towards virus (79, 80). Therefore, STING agonists are promising adjuvant for constructing effective SARS-CoV-2 vaccines.

cGAMP, the natural ligand of STING, has been widely investigated as adjuvant in vaccine development. cGAMP and S protein-loaded HIV-derived virus-like particles (VLPs) was reported to induce more potential antibody response compared with S protein-loaded VLPs. The virus neutralizing capacity of resulting antibodies was improved as well (81). An intranasal subunit vaccine accompanied by liposomal cGAMP and lyophilized S protein was reported to trigger robust neutralizing antibodies and comprehensive immune response in lung, spleen and nasal compartments (82). Additionally, a ternary adjuvant system consisting of Alum, cGAMP and TLR3 agonist poly(I:C) was used to construct a S1 protein vaccine, and the ternary adjuvant showed potent adjuvant effect on inducing humoral and cellular immunity without apparent biological toxicity in immunized mice (83).

In addition to cGAMP, a few novel STING agonists were also used as adjuvants in SARS-CoV-2 vaccine. For instance, Wu and co-workers synthesized the analogue CDGSF by modifying cyclic di-GMP (CDG) with one phosphorothioate and one fluorine moieties (84). The fluorine modification enhanced the liposolubility and stability of CDG (85), and increased the expression of CD86 in macrophage and bone marrow-derived dendritic cells (BMDC). As adjuvant, CDGSF significantly improved the S protein-specific IFN-γ secretion and IgG titers, more potent than classical adjuvant Alum, thus highlighting the adjuvant potential of CDGSF in SARS-CoV-2 vaccine preparation.

CF501 is a new STING agonist and was found to show potent adjuvant efficacy in *pan*-sarbecovirus vaccine (86). Compared with Alum- or cGAMP-adjuvanted RBD-Fc-based vaccines, intramuscular injection of CF501-adjuvanted RBD-Fc vaccine (CF501/RBD-Fc) triggered stronger humoral and cellular immune responses against various variants of SARS-CoV-2, SARS-CoV and SARSs-CoVs from bats in mice, rabbit and rhesus macaques models. Further, CF501/RBD-Fc induced long-term protective immunity against SARS-CoV-2 challenge in both macaques and hACE-transgenic mice. Moreover, CF501 transiently triggered innate immunity without obvious lesion in the tissue of CF501/RBD-Fc-immunized mice, suggesting the good safety profile of CF501 as adjuvant (86).

## Discussion

**Figure 2:**
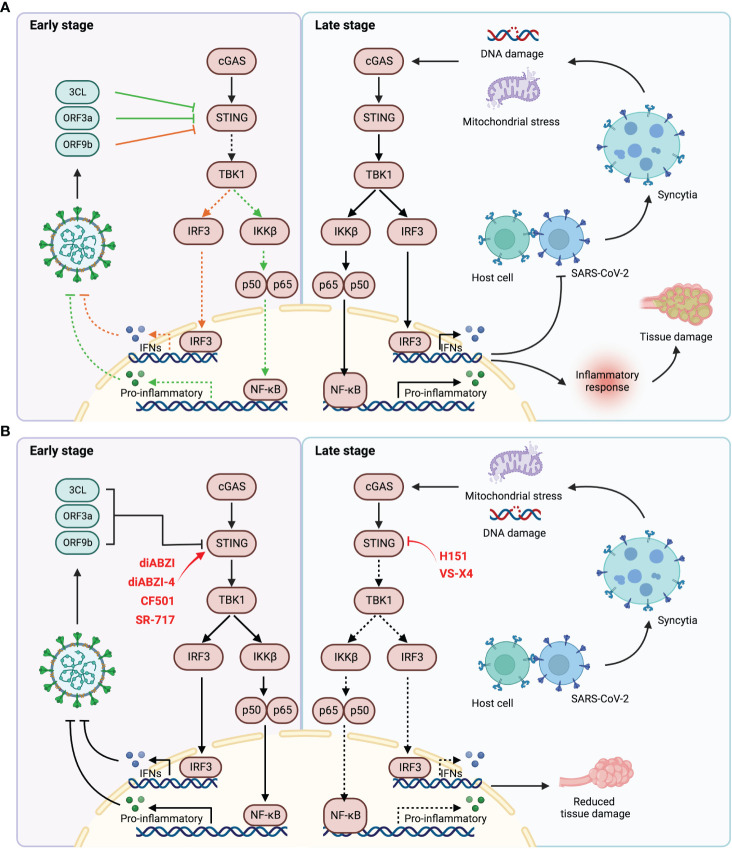
The relationship of SARS-CoV-2 infection and STING pathway. **(A)** At the early stage of SARS-CoV-2 infection, the viral proteins inhibited the activation of STING pathway by direct interaction with STING (left). During the late stage of infection, the host cells fused with virus through the interaction of ACE2 and S protein to form syncytia, which contains a large number of micronuclei, mediating DNA damage and thus activating STING signaling. Durable and excessive STING activation lead to abnormal inflammatory response, resulting in tissue damage and poor prognosis (right). **(B)** STING agonists could be used to activate STING signaling at the early stage of infection to elicit anti-viral response (left). STING inhibitors could attenuate tissue damage by suppressing excessive STING activation and aberrant inflammatory response (right).

It is well established that the STING pathway elucidates a double-edged effect on COVID-19. At the early stage of infection, STING signaling is suppressed by vital proteins containing 3CL, ORF3a and ORF9b, resulting in the impaired innate immune response (38, 41). In contrast, the fusion of S protein of the virus with the ACE2 receptor in host cells leads to syncytia formation, resulting in formation of micronucleus and DNA damage, and consequently triggering the STING signaling and antiviral response (50, 51). These may account for the observed activation delay of type I IFN response in COVID-19 patients (57). Therefore, treatment with STING agonists could effectively activate innate immune response to inhibit virus infection and replication. However, excessive and sustained activation of STING signaling leads to accumulation of inflammatory factors, resulting in abnormal lung inflammation and poor clinical outcomes (**Figure 2**) (52, 58, 64). Hence, trials of STING agonists and inhibitors in the treatment of COVID-19 should be cautiously evaluated in context. Timing and duration are critical factors, at the early state of infection, STING agonists may be used to recuse the deficiency of IFNs production. Instead, the inhibitors of STING could be applied to suppress the excessive inflammatory response and to alleviate the tissue injury caused by the disease. Since type I IFN response is the bridge between STING pathway and COVID-19 progression, we speculate that the content of interferons in the patients might be a bio-marker for the use of STING regulators in clinical.

## Author contributions

XR collated the literature and wrote the review, and ZA established that the structure of the review and revised the article.
